# Surgery as a Principle and Technical Consideration for Primary Tumor Resection of Small Bowel Neuroendocrine Tumors

**DOI:** 10.1245/s10434-023-14610-0

**Published:** 2023-11-25

**Authors:** Kjetil Søreide, Stefan Stättner, Julie Hallet

**Affiliations:** 1 Department of Gastrointestinal Surgery, Stavanger University Hospital, Stavanger, Norway; 2 Gastrointestinal Translational Research Group, Laboratory for Molecular Medicine, Stavanger University Hospital, Stavanger, Norway; 3 Department of Clinical Medicine, University of Bergen, Bergen, Norway; 4 Department of General, Visceral and Vascular Surgery, Vöcklabruck, Austria; 5 Department of Surgery, University of Toronto, Toronto, ON Canada; 6 Susan Leslie Clinic for Neuroendocrine Tumors – Sunnybrook Health Sciences Centre, Toronto, ON Canada

## Abstract

Small bowel neuroendocrine tumors (SB-NETs) are increasingly identified and have become the most frequent entity among small bowel tumors. An increasing incidence, a high prevalence, and a prolonged survival with optimal modern multidisciplinary management makes SB-NETs a unique set of tumors to consider for surgical oncologists. The major goals of surgical treatment in the setting of SB-NET include control of tumor volume, control of endocrine secretion, and prevention of locoregional complications. Key considerations include assessment of multifocality and resection of mesenteric nodal masses with the use of mesenteric-sparing approaches and acceptance of R1 margins if necessary to clear disease while avoiding short bowel syndrome. A description through eight steps for consideration is presented to allow for systematic surgical planning and execution of resection. Moreover, some controversies and evolving considerations to the surgical principles and technical procedures remain. The role of primary tumor resection in the presence of (unresectable) liver metastasis is still unclear. Reports of feasibility of minimally invasive surgery are emerging, with undetermined selection criteria for appropriateness or long-term outcomes. Resection of SB-NETs should be considered in all patients fit for surgery and should follow principles to achieve surgical oncological control that is appropriate for the stage and tumor burden, considering the age and comorbidity of the individual patient.

Small bowel neuroendocrine tumors (SB-NETs) were first described by Otto Lubarsch in 1888^1^ and only a few decades later were named ‘carcinoids’ by Sigfried Oberndorfer based on the cancer-like morphology of the cells in the tumor.^2^ However, due to the unprecise terminology, these lesions were renamed ‘neuroendocrine tumors’ in 2010. Collectively, such tumors in the gastrointestinal tract are known as gastroenteropancreatic neuroendocrine neoplasia (GEP-NEN).^3,4^ GEP-NEN constitute a heterogeneous group of neuroendocrine tumors (NETs) or neuroendocrine cancers (NECs) with largely different biological and clinical behavior, and classified according to the updated WHO 2019 recommendations (Table 1).^5^

**Table 1:** WHO 2019 classification for GEP-NEN

Grade	Mitotic count (2 mm^2^)^a^	Ki-67 index (%)^b^	Morphology
G1	< 2	< 3	Well-differentiated NETs
G2	2–20	3–20	Well-differentiated NETs
G3	> 20	> 20	Well-differentiated NETs
NEC	> 20	> 20	Poorly differentiated NECs

*GEP-NEN* gastroenteropancreatic neuroendocrine neoplasia, *NECs* neuroendocrine carcinomas, *NETs* neuroendocrine tumors, *WHO* World Health Organization, *HPF* high-power fields

^a^10 HPF = 2 mm^2^, at least 40 fields (at ×40 magnification) evaluated in areas of highest mitotic density

^b^MIB1 antibody; percentage of 500–2000 tumor cells in areas of highest nuclear labeling

The rising incidence and accumulating prevalence in the population makes GEP-NEN a common and clinically relevant disease.^6–10^ While each separate location may represent somewhat rare diseases, all GEP-NENs taken together are estimated to be the second most prevalent tumor in the digestive tract (after colorectal cancers) in terms of prevalence.^11^ Among all GEP-NENs, the small bowel represents one of the most common tumor locations.^7,8,12–14^ The combination of a rising incidence, a high prevalence and prolonged survival make this group of patients represent a ‘chronic’ cancer condition with unique needs and opportunities for treatment for which surgery represents an essential part.

Surgery has a major role in the management of patients with SB-NETs, as declared in European Neuroendocrine Tumor Society (ENETS) consensus guidelines from 2016^15^ and 2017,^16^ and the North American Neuroendocrine Tumor Society (NANETS) consensus guidelines from 2017.^17^ The major goals of surgical treatment in the setting of SB-NETs include control of tumor volume, control of endocrine secretion, and prevention of locoregional complications. However, some controversies and evolving considerations to the surgical principles and technical procedures remain for consideration.^18–22^ As surgical principles and understanding evolve with updated diagnostic criteria, disease understanding and novel adjunct treatment modalities, there is a need for updated knowledge and considerations to surgical approaches and principles for SB-NET.

In this review, we focus on the role of surgical resection for SB-NETs and consider the current and debated principles of surgical oncological management of this condition to aim at cure, prevent progressive complications, and alleviate symptoms.

## Clinical Presentation

Due to the untypical and often diffuse symptomatology, SB-NETs may present with a considerable delay from onset of symptoms to eventual diagnosis. SB-NETs are characterized by slow, indolent growth and non-specific symptoms. The associated delay in diagnosis often results in advanced stage at presentation, with up to 30% of patients having metastases at the time of diagnosis.^9,23^ Notably, both locoregional and metastatic disease can severely impair quality of life for patients with SB-NETs.

**Fig. 1:**
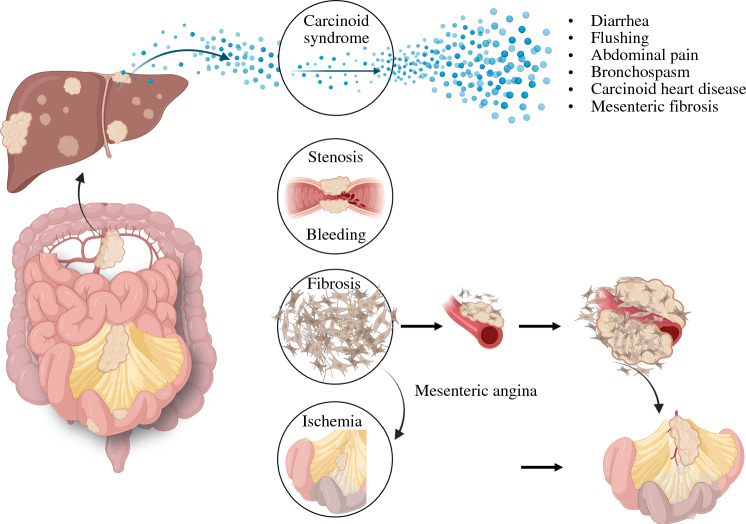
Symptoms and complications associated with disease mechanism and progression in small bowel neuroendocrine tumors. Figures made in part by elements from Biorender.com

Locoregional disease may manifest with chronic abdominal pain from mesenteric angina, intermittent gastrointestinal obstructive symptoms, or ureteral obstruction, related to desmoplastic reaction that is present in 50% of patients with SB-NETs;^24–26^ ultimately, this evolves towards malabsorption, malnutrition, and cachexia (Fig. 1).^25,26^ Some 35% of patients may also present with acute onset symptoms that leads to diagnosis of SB-NET, with or without prior evolving chronic symptoms.^27^ Importantly, the level of locoregional fibrosis and associated symptoms is not related to systemic serotonin (or measurable urinary 5-HIAA). Indeed, mesenteric (or retroperitoneal) fibrosis appears related to changes in the local microenvironment; even small levels of fibrosis or mild progression of fibrosis can create considerable symptoms interfering with quality of life.^25,28^ Because such fibrosis does not reduce overall survival, it can have lasting detrimental impact for many years.^28^ Importantly, fibrosis and its repercussions are mostly related to the nodal metastases rather than the primary SB-NET itself.

When assessing symptoms from locoregional disease, it is important to keep in mind that patients tend to cope with slowly evolving symptoms due to indolent courses of NET growth. Symptomatic status for SB-NETs is difficult to ascertain accurately. Patients get used to insidious abdominal symptoms often for years prior to NETs diagnosis; however, data indicate that patients with NETs have multiple investigations and clinical assessments prior to diagnosis, which suggest that they had symptoms warranting medical attention often long before diagnosis.^15,17^ In fact, in historical series, up to 80% ‘retrospective appreciation’ of symptomatic relief has been reported following SB-NET primary tumor resection.^29,30^

For metastatic disease, the manifestations of carcinoid syndrome are well-described, including flushing, diarrhea, wheezing, and, eventually, carcinoid heart disease.^31^ Carcinoid syndrome may occur in up to 20% of all patients with NETs, while small bowel location of the primary is associated with carcinoid syndrome in up to 40%.^32^ Similar to locoregional symptoms, carcinoid syndrome-related symptoms evolved over years and patients can cope with them over time. Those symptoms can therefore be subtle upon clinical assessment. It is important to obtain biochemical endocrine assessment with 24-h urinary 5-hydroxyindoleacetic acid (5-HIAA) for all patients with SB-NETs; many instances of serotonin hypersecretion can only be detected this way. In particular, carcinoid heart disease (the end result of long-lasting carcinoid syndrome) occurs in 4% of patients with neuroendocrine neoplasms but 40% of those with carcinoid syndrome. It can often go undetected such that investigation with echocardiogram is crucial in patients with metastatic SB-NETs,^33^ elevated 24-h urinary 5-HIAA, or carcinoid syndrome symptoms.

## Diagnostic Work-Up and Staging

Staging for SB-NET should involve (1) identification of the primary site; (2) structural tumoral staging; and (3) endocrine staging. Identification of the primary site refers to confirmation of a primary SB-NET and assessing the extent, as both locoregional and multifocal disease; this involves both cross-sectional imaging and functional imaging. Structural tumoral staging refers to traditional cancer staging for identification of distant metastases and involves both cross-sectional imaging and functional imaging. Finally, endocrine staging involves identification of functional tumors and associated endocrine syndromes and their repercussions; it involves measurement of serotonin production with 24-h urinary 5-HIAA and echocardiogram.

The specific indications, sensitivity, and role of imaging modalities are reported extensively by expert panels.^34^ Contrast-enhanced, cross-sectional imaging may suffice to pursue surgical resection for some patients presenting urgently with small bowel obstruction or symptomatic/emergency presentation indicating a small bowel tumor. In most other situations, decision making is based on a proper diagnosis based on cross-sectional imaging by means of a multiphase, contrast-enhanced computed tomography (CT) with arterial-phase contrast, as NETs are typically enhancing in the arterial phase due to their hypervascularity.

Magnetic resonance imaging (MRI) scanning with liver-enhanced contrast (e.g., Eovist, Primovist) for evaluation of diffusion-weight imaging^35^ may be pursued if there are suspicious lesions or presence of liver metastasis. Contrast-enhanced MRI provides a more detailed assessment of the liver metastatic burden and directly contributes to surgical assessment and planning. By knowing the full extent of the metastases, it is easier to plan maximal cytoreduction with a combination of liver parenchymal-sparing resections and ablations, as deemed necessary.

**Fig. 2:**
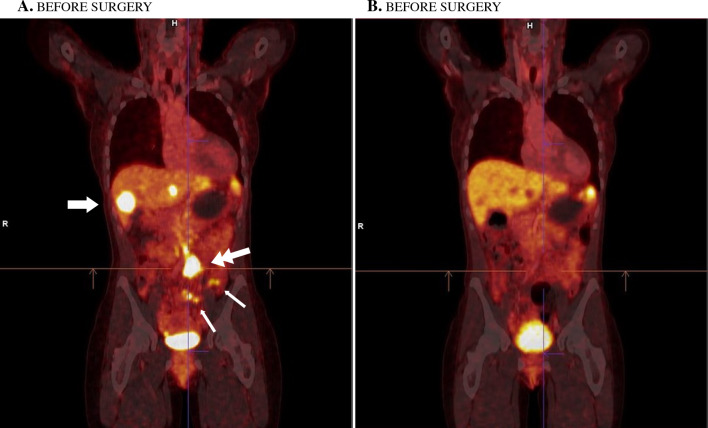
Gallium-PET in locally advanced and metastatic SB-NETs. **A** Before surgery, showing high uptake in small bowel lesions (smaller arrows), large mesenteric deposit (double arrow), and liver metastasis (bold arrow). Background signal present in the spleen. **B** After surgery, with only a physiological signal in the spleen and urinary bladder. *PET* positron emission tomography, *SB-NETs* small bowel neuroendocrine tumors

Specific functional imaging tests in diagnosis and staging has an increasing role and superior sensitivity in most settings for SB-NETs.^34,36,37^ Overall, these tests are known as somatostatin receptor PET imaging (SSTR-PET) and include several types of tracers.^34^ As such, functional imaging may include somatostatin receptor scintigraphy (now largely replaced by more specific PET tracers), ^68^Gallium-PET and ^18^FDG-PET, with more novel tracers in use per institutional availability (Fig. 2).^35,38,39^ For high-grade NET (e.g., Ki-67 proliferation index > 15%) and NEC,^40^ the sensitivity is considered better for FDG-PET, and hence FDG-PET is preferred due to a higher glucose uptake for these often overt malignant tumors.^41–43^

Of note, G3 NETs and NEC are rather unusual for SB-NETs (< 1%). With SSTR-PET, the higher the uptake (or SUV) of a lesion, the higher the concentration of somatostatin receptor, hence the higher the differentiation and the better the biology; this is a different interpretation for SUV than for better-known FDG-PET imaging. SSTR-PET imaging for SB-NETs can help in the following clinical scenarios: (1) confirm diagnosis of NETs when the mesenteric mass or small bowel lesion is not amenable to biopsy; (2) investigation of occult primary tumor after CT or MR enterography; (3) when considering cytoreduction surgery, to ensure all maximal cytoreduction is planned; (4) when borderline indication for cytoreduction and identification of additional disease would contraindicate surgery; and (5) to assess intra- or inter-tumor biology heterogeneity if it will alter management. With regard to diagnosis of NETs, avidity on SSTR-PET can be considered diagnostic of NET; the level and pattern of avidity can also reflect tumor grade.^44,45^

CT or MR enterography is also warranted to assess the full extent of SB-NETs, or detect tumors labeled as ‘unknown primaries’,^34^ as the latter may occur in 10–20% of patients presenting with neuroendocrine liver metastasis.^46^ Multifocal SB-NETs will occur in up to 56% of patients,^47^ with tumors most often located in the distal 100 cm of the small bowel.^48^

When liver metastases or mesenteric masses are identified with no evidence of a primary tumor, immunohistochemistry and enterography studies can most often identify the primary SB-NETs. Staining for CDX2 points toward an SB primary.^49^ CT enterography and MR enterography have sensitivity of 76% and 93%, respectively, for detecting occult SB-NETs that are often small compared with the mesenteric mass or the distant metastases they are associated with. When using CT or MR enterography, it is important to consider the expertise of the radiology technician performing the study (regarding timing of image acquisition and the radiologist reading it), as they can affect the accuracy of the examination.

A definitive diagnosis of any NET should be attempted by a core biopsy (if not proceeding to surgery) or surgical resection for histological evaluation, as fine needle aspiration or cytology is insufficient for a specific diagnosis (use of immunohistochemical markers) and evaluation of proliferation (mitosis or Ki-67%). The updated World Health Organization (WHO) 2019 classification separates NENs into four grades (Table 1). Surgery is indicated for all G1 and G2 NETs, but is more controversial in higher grades (NET G3) and is usually contraindicated in NEC. Clinical TNM classification and stage is presented in Table 2.^5^

**Table 2:** TNM clinical classification for SB-NETs (WHO 2019)

T—Primary tumor^a^			
Tx Primary tumor cannot be assessed			
T0 No evidence of primary tumor			
T1 Tumor invades mucosa or submucosa and size ≤ 1 cm in the greatest dimension			
T2 Tumor invades muscularis propria or size > 1 cm			
T3 Tumor invades through the muscularis propria into subserosal tissue without penetration of overlying serosa (jejunal or ileal)			
T4 Tumor perforates visceral peritoneum (serosa) or invades adjacent structures or other organs			
N—Regional lymph node metastasis			
Nx Regional lymph nodes cannot be assessed			
N0 No regional lymph node metastasis			
N1 < 12 regional lymph node metastasis without mesenteric mass(es) > 2 cm in size			
N2 ≥ 12 regional lymph node metastasis and/or mesenteric mass(es) > 2 cm in maximum dimension			
M—Distant metastasis			
Mx Distant metastasis cannot be assessed			
M0 No distant metastasis			
M1 Distant metastasis			
M1a Hepatic metastasis only			
M1b Extrahepatic metastasis only			
M1c Hepatic and extrahepatic metastasis			

Stage I	T1	N0	M0
Stage II	T2, T3	N0	M0
Stage III	T4 Any T	Any T N1, N2	M0 M0
Stage IV	Any T	Any N	M1

*SB-NETs* small bowel neuroendocrine tumors, *WHO* World Health Organization

^a^For any T, add (m) for multiple tumors

## Preoperative Classification of Small Bowel Neuroendocrine Tumors (SB-NETs)

A major goal with preoperative imaging is to plan treatment sequencing and allow for optimal preoperative planning.^50^ Of note, all microscopic disease will not be detected by current methods.^51,52^ Tumor mass clearly visible on a CT scan may allow for a planned surgical strategy, e.g., the likelihood of minor or major resection involved, or even if debulking is the only possible option. Hence, a triphasic CT scan (for locoregional tumor staging), contrast-enhanced MRI (for liver metastases) and ^68^Ga-DOTATOC PET scans (for additional hepatic/extrahepatic disease) will allow for the most detailed preoperative planning. Of note, not all disease will be visible on imaging, hence the need for exploration and palpation.

**Fig. 3:**
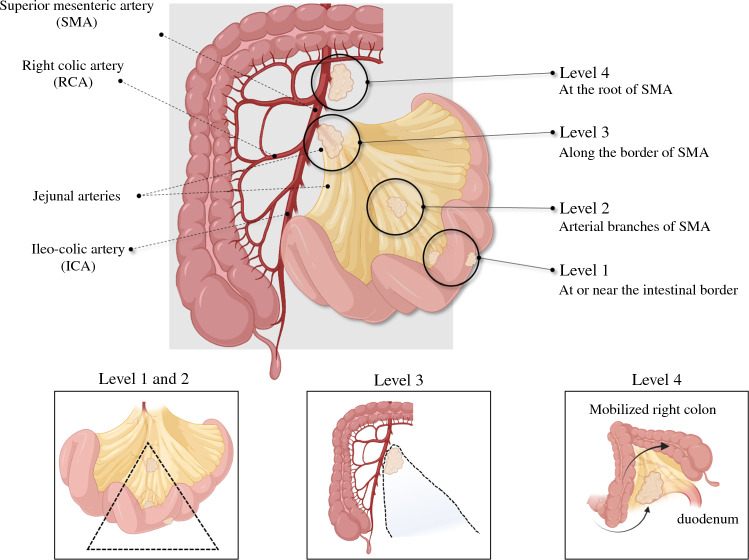
Four-level classification of mesenteric masses in SB-NETs. Level 1 is close to or at the intestinal border; level 2 node/mass is sitting on the arterial branches from the SMA; level 3 is located along the border of the SMA; and level 4 extends in the retroperitoneum to the root of the SMA (at or under the pancreatic neck). Inserts at the bottom suggest surgical management strategies associated with the level of mesenteric mass (see text for further details). *SB-NETs* small bowel neuroendocrine tumors, *SMA* superior mesenteric artery. Figures made in part by elements from Biorender.com

SB-NET mesenteric masses are categorized in four levels (Fig. 3), as proposed by Ohrvall and colleagues.^30^ The original four-level system has later been suggested to be modified to three levels,^22^ but most studies report a four-level definition, as presented in this review (Fig. 3). Level 1 is near the intestinal border, level 2 is sitting on arterial branches from the superior mesenteric artery (SMA), level 3 is located along the border of the SMA, and level 4 extends in the retroperitoneum to the root of the SMA (at or under the pancreatic neck). A detailed assessment of which arterial and venous mesenteric branches may be involved or abutted by tumor is critical in the decision making and surgical planning for lesions visible on imaging.

## Principles of Primary Tumor Resection

The goals of management of the primary tumor resection in SB-NETs are to provide tumor control (whether for curative-intent resection or for cytoreduction depending on the extent of disease) and endocrine control, but also to relieve symptoms and/or prevent complications from fibrosis that can impair quality of life, such as obstruction, mesenteric angina, and gastrointestinal bleeding.^21,53–55^

When determining resectability, it is usually not the primary tumor of the small bowel (e.g., a T4-stage setting with invasion of other organs) that results in technical unresectable disease but rather the extent of the mesenteric deposits and level of lymph node involvement.^16,56^ Hence, resectability has been classified according to types A, B and C, whereby type A is an SB-NET with resectable mesenteric disease (including both lymph node metastases and associated fibrosis) that does not involve the mesenteric root, including the origin of the SMA; type B is a ‘borderline resectable’ SB-NET presenting with mesenteric nodal metastases and fibrosis adjacent to the main trunk of the SMA and superior mesenteric vein (SMV) but not encasing the vessels; and type C is ‘locally advanced or irresectable’ SB-NET where tumor deposits and fibrosis encase the SMA and SMV.^56^ This proposed classification largely overlaps the four-tier system proposed by Ohrvall and colleagues,^30^ as depicted in Fig. 3.

It is important to keep in mind the long-term survival of patients with SB-NETs, with survival at 10 years despite the fact that a high rate of patients eventually develop metastases. In SB-NETs, small bowel resection can be performed with low morbidity and mortality, with 30-day mortality and morbidity reported well below 2% and 20%, respectively.^57–59^

## Role of Exploration for Unifocal or Multifocal Tumors

**Fig. 4:**
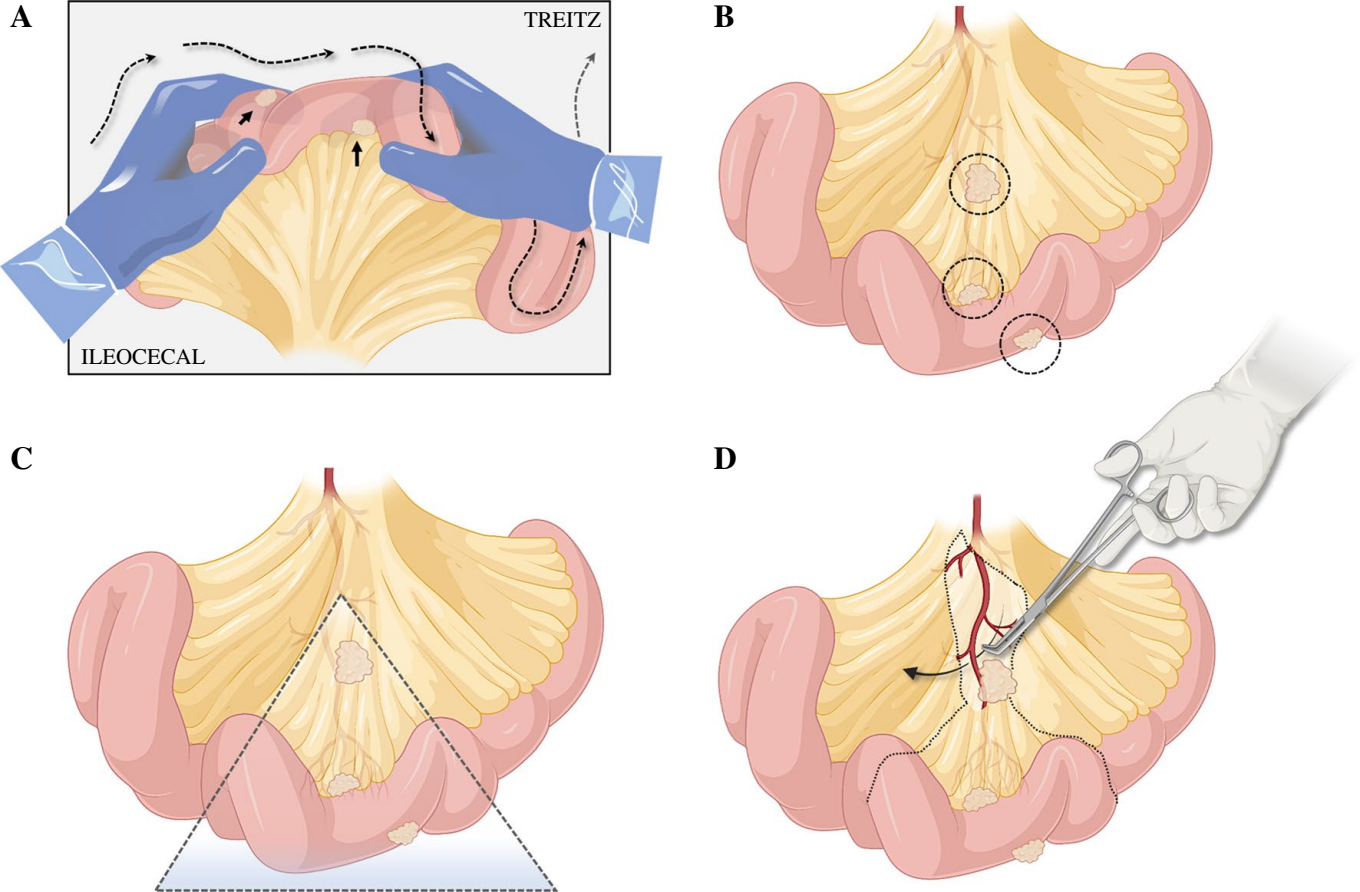
Principles of SB-NET identification and determination of the level of resection. **A** Systematic bi-manual palpation from the ileocecal valve to the ligament of Treitz. **B** Identification of SB-NET lesions in the small bowel and the mesentery. **C** Determination of an outline for resection. **D** Performing a vessel-sparing and mesentery-sparing approach. *SB-NETs* small bowel neuroendocrine tumors. Figures made in part by elements from Biorender.com

Because of the high risk of multiplicity in SB-NETs, an open laparotomy that allows for full exploration of the abdomen and running the entire small bowel is preferred as a reference standard. Bimanual palpation (Fig. 4a) should be performed systematically from the ileocecal valve to the ligament of Treitz. This is a good opportunity to utilize both the operating surgeon and the assistant surgeon’s hands and eyes (‘four hands and four eyes’ approach) to detect any small lesions not seen on imaging, even if the investigation by one experienced surgeon may suffice. In a large Scandinavian cohort, almost 40% of patients had more than one SB-NET at the time of diagnosis.^47^ Most patients had multifocality of primary tumors before metastasis developed, making this a particular feature of NETs, as the multifocality needs to be recognized during surgery and may lead to extensive resections. A bowel-sparing strategy is essential to avoid short bowel syndrome from extensive resections.

A systematic approach to the number, location, level of the tumors (Figs. 3 and 4), lymph nodes and tumor deposits in the mesentery should be made, and use of proper surgical planning for resection (Fig. 4b–d). For levels I and II, traditional small bowel resection and lymph node dissection can be undertaken. For levels III and IV, a mesenteric-sparing approach is favored to allow for resection for complex proximal nodal masses while preserving intestinal length and function. Caution should be advised for attempting resection of proximal nodal masses that involve the mesenteric vessels and extend to the root of these vessels. Lesions that encase the mesenteric vessels in this place are at high risk for vascular injury and compromise of vascular inflow and outflow if overzealous resection is attempted. Notably, patients may live on for years (reported at 7–8 years as a median for resected SB-NETs) with such lesions, and even if vessels are compromised causing venous congestion, collaterals may develop and reduce symptoms over time. Each case needs to be tailored to the risk and benefit for when to perform extra lymphadenectomy for nodes and masses not included in the resected bowel. Easily resected nodes that are round and discrete can be taken off of involved vessels and may be dissected circumferentially in the mesentery, sparing as much bowel as possible.

As tumors are encountered, marking stitches can be placed at the level of the most proximal and most distal lesions. The length of intestinal involved and uninvolved by tumors should be measured and documented; free ties can be used to do so as they have the advantaged of adapting to the shape of the intestine, which rulers cannot easily do. These measurements, as well as the pattern of mesenteric nodal masses, will dictate the type of resection. A single resection is favored if feasible with sufficient remnant small intestinal length. In the case of multifocal tumors, if the resection with complete lymph node dissection leads to loss of extensive intestinal length, a more limited mesenteric resection can be undertaken to ensure nodal harvest while preserving intestinal function. In addition, efforts should be made to preserve the ileocecal artery (and, as a result, the ileocecal valve); to this end, anastomoses on the last 10 cm of the terminal ileum can be performed. Both those considerations are crucial in the case of carcinoid syndrome or subsequent need for re-resection in the case of recurrence (up to 60% at 15 years).^60^ The length of residual small intestine should be documented (and aimed for length above 200 cm to avoid short bowel syndrome), as this information will be important in the long-term management of the NETs.

## Surgical Prevention of Mesenteric Fibrosis

Goals of care for SB-NETs and management of the primary tumor depend on the extent of disease. In general, focus is on control of the tumor burden, control of the endocrine secretion, and prevention of complications that could impair quality of life, such as obstruction, mesenteric angina, and gastrointestinal bleeding.^53–55^ For patients with locoregional SB-NET tumors, about half of them will develop mesenteric and/or retroperitoneal fibrosis due to the fibroblastic reaction (Fig. 1) surrounding the primary tumor and nodal metastases.^25,26^ Such fibrosis can lead to mesenteric angina and ischemia or venous congestion of vessels. Fibrosis may also cause partial or complete bowel obstruction. Ultimately, worsening fibrosis may lead to chronic abdominal pain, malabsorption, malnutrition, and cachexia.^28^

Locoregional SB-NETs can be treated with curative intent, with risk of recurrence, while metastatic SB-NETs can rarely be cured even if they can be treated with prolonged survival with persistent disease.^54,59,61,62^ In the latter setting, surgical palliation or pre-emptive surgery to avoid future complications (i.e., mesenteric stenosis or bowel ischemia) may be indicated, although this is still debated for its impact on overall survival.

## Locoregional SB-NETs with Clinically Negative Nodal Disease

Resection of the primary tumor along with regional lymph nodes to target resection of ≥ 8 lymph nodes for staging is the standard of care for curative intent.^63,64^

The extent of bowel resection depends on the number and location of potentially multifocal SB-NETs (Fig. 3). With clinically negative nodal disease, lymph node dissection aims for the identification of microscopic nodal metastases for staging and prevention of growth and subsequent fibrosis symptoms.^60^ The lymphadenectomy is performed for staging and prevention of recurrent nodal disease and associated fibrosis. In patients with multifocal tumors at risk for loss of extensive intestinal length by aggressive surgery, a more limited mesenteric resection should be undertaken to ensure nodal harvest while preserving intestinal function. In addition, efforts should be made to preserve the ileocecal artery. Both these considerations are crucial in the case of carcinoid syndrome or subsequent need for re-resection in the case of recurrence (up to 60% at 15 years).^60^ The decision between aggressive, curative-approach resection must be balanced with the risk of loss of bowel length that may lead to short bowel syndrome and poor quality of life in the individual patient.

## Locoregional SB-NETs with Clinically Positive Nodal Disease

Overall, almost 50% of patients with SB-NETs will present with nodal mesenteric masses at the time of diagnosis.^47,63^ For patients with locoregional, clinically node-positive SB-NETs, every attempt should be made at resection of the primary tumor together with the nodal mesenteric mass for a curative-intent approach and to prevent future debilitating complications from developing mesenteric fibrosis. As many as one in every two patients with mesenteric nodal masses present with abdominal pain or intestinal obstruction, and resection of the mesenteric mass can provide relief of symptoms and prolonged survival.^12,59,61^ When the nodal mass is large or extending proximally along the axis of the SMA (such as level 2 and 3 masses), a mesenteric-sparing resection may be needed to resect the bulk of the mass. An R1 margin is then accepted, considering the need to prevent debilitating fibrosis-related complications while avoiding short bowel syndrome and the indolent growth of NETs, making recurrence at the R1 margin unlikely or extremely low over the course of disease.^62,65^

Small bowel resection in a mesenteric-sparing manner allows for resection of proximal nodal masses to prevent complications related to the desmoplastic reaction while avoiding short bowel syndrome from too aggressive surgery.^62,65^ As such, all patients with SB-NETs with nodal mesenteric mass should be assessed by experienced NET surgeons to confirm the feasibility of proximal mesenteric-sparing resection.

## Technical Considerations in Surgical Resection of SB-NETs

Surgery for SB-NETs should follow a standard set of principles, although a tailored approach must be made for each individual patient based on preoperative staging, symptoms and urgency of presentation, as well as age and associated comorbidity. A mesenteric-sparing approach should be favored, as short bowel syndrome is an unwanted and debilitating consequence of too extensive resections of the small bowel. Some key steps for mesenteric-sparing resection have evolved over the years and should essentially follow a structured plan.^62^ A structured, stepwise approach should be applied after appropriate preoperative planning and thoughtful consideration of how these will align to the surgical principles outlined.^17,18,20,22,66^

Careful preoperative examination of the imaging to identify involvement of the mesenteric branches is key. This obviously relates to any identifiable, visible lesion on radiological imaging, as non-visible (but palpable on surgery) lesions are expected to be discovered during surgery. There should be a minimum of two arterial and venous mesenteric branches/tributaries either free or that can be freed of tumor for proximal mesenteric-sparing resection to be feasible. When there is contact between the mesenteric nodal mass and arterial approaches, a ‘smooth’ appearance (round, discrete node/mass) indicates that the mass may be dissected off the vessel, whereas a ‘scalloped’ appearance indicates the likelihood of involvement of adventitia and dissection of that vessel will not be possible.

*Step 1:* Complete exploration of the abdomen focused on the entirety of the small bowel (palpation from the ileocecal valve to the ligament of Treitz, as depicted in Fig. 4a), including the liver, omentum, and pelvis. The exploration includes localization of any small bowel tumor(s) [Fig. 4b] and/or the corresponding mesenteric mass(es).

*Step 2:* This step should facilitate complete mobilization of the involved mesentery off of the retroperitoneum to allow for increased length of the mesentery and releasing of the mesenteric mass from the surrounding retroperitoneal structures, such as the duodenum and pancreas. Attention should also be paid to lesions on the diaphragm and organs in the pelvis such as the rectosigmoid and bladder and ovary in women, with the aim to excise any palpable or visible lesion. With the mobilization, the mesenteric mass will lower and the true location on the mesenteric axis will be clearer, and manipulation with anterior and posterior aspects will make dissection easier. Some surgeons include a complete Cattell–Braasch maneuver at this step if needed, but this usually only applies to level 4, and sometimes level 3, tumors (Fig. 3).

*Step 3:* Initiation of resection of the mesenteric nodal mass by incising the anterior peritoneum at the proximal aspect of the mass. Dissection then begins on the edge of the nodal mass to free it from underlying mesenteric vessels. This step should release the desmoplastic fibrotic reaction and lengthen the mesentery, and hence further releasing the mass from the central vessels (Fig. 4d).

*Step 4:* In this step, intramesenteric dissection is done to identify and free the vascular pedicles proximal to the mesenteric involvement and to allow understanding of the vascular anatomy in relation to the mesenteric mass. It is important to dissect and preserve both arterial and venous branches. As previously mentioned, in these complex circumstances, an R1 margin is accepted if the lesion can be peeled off of the vessel.

*Step 5:* The extent of small bowel required for resection based on the planned vascular transection level must be carefully assessed. Usually this is straightforward if the transection is set at the level I–II lymph nodes (Figs. 3 and 4c). For patients with SB-NET with level III lymph nodes involved, a more extensive intramesenteric and retroperitoneal dissection may be required. Test clamping (i.e., with bulldog clamps) of vascular pedicles at the anticipated level of transection can be done to assess the level of demarcation in the proximal and distal small bowel. The extent of resected bowel, and that which is remaining, is measured.

While radical surgery is the aim to remove as much tumor load as possible, care should be taken to spare as much small bowel as possible. A remaining small bowel length in continuity of < 200 cm from the ligament of Treitz is strongly associated with the risk of developing short bowel syndrome,^67^ and is associated with malabsorption, diarrhea, fatty stools, malnutrition, and dehydration. Short bowel syndrome may severely impact both quality of life and overall survival per se.

*Step 6:* Isolation and transection of the vascular pedicle (Fig. 4d). The vascular pedicle can be isolated using a drain or vascular sling and transected by a vascular stapler or suture ligature or clips (e.g., Hem-O-Lock), as preferred or available. Sometimes the transection may need to be performed flush on the tumor-bearing vessel to allow for preservation of nearby critical vessels. A compromise between radical R0 and a debulking R1 may sometimes be needed in order to not compromise the remaining length of bowel (to avoid short bowel syndrome from developing).

*Step 7:* Transection of the more peripheral mesentery around the area of disease will then follow. This can be done sharply with ligatures or with the use of an energy device of choice (i.e., LigaSure or Harmonic scalpel) around the mass and towards the bowel wall on the proximal and distal side of the mesentery.

**Fig. 5:**
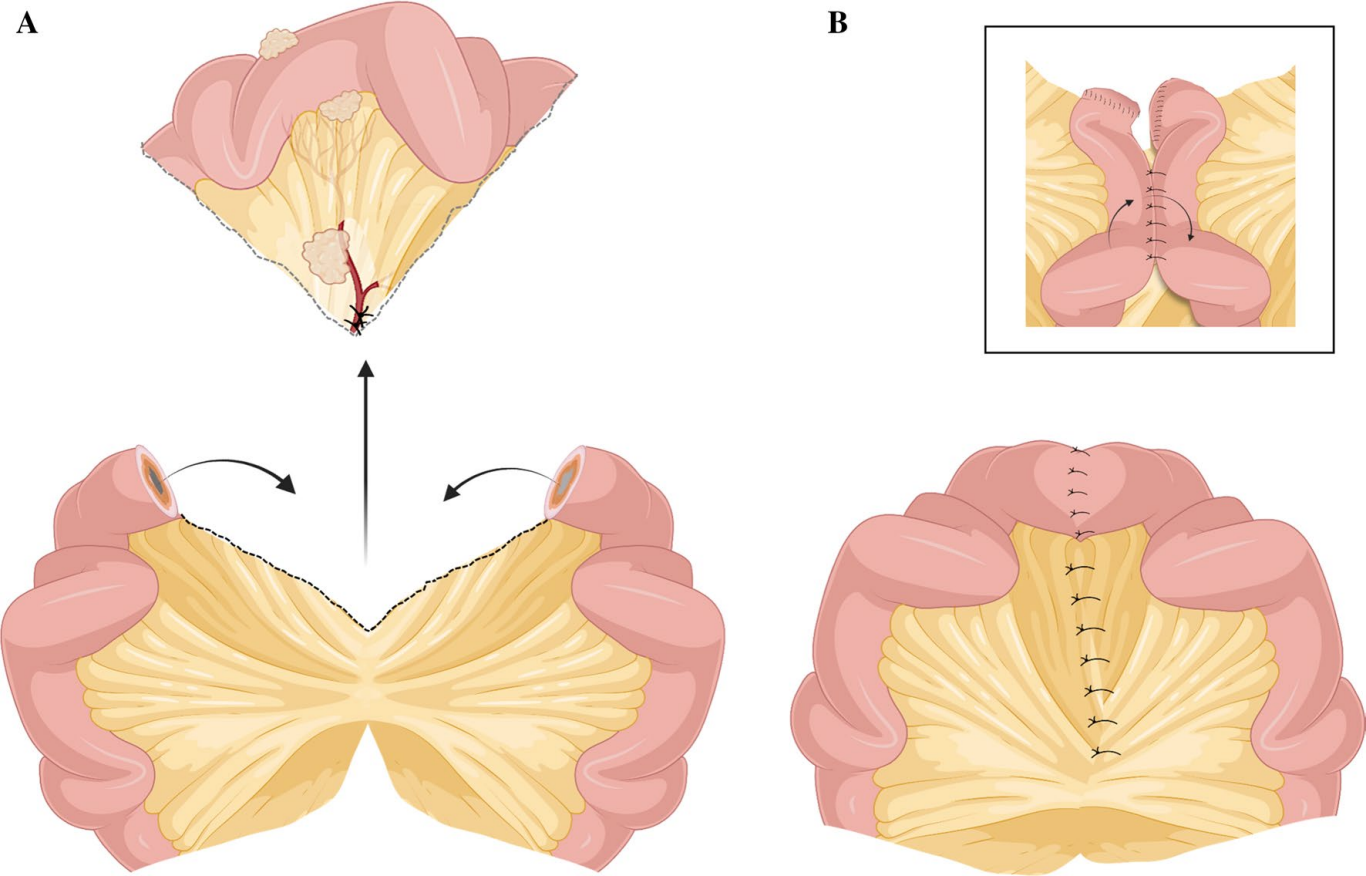
Resection of the SB-NET segment and reconstruction of small bowel continuity. **A** The resected specimen is removed for histopathological analyses and tumor grading. **B** Reconstruction, either as an end-to-end or side-to-side entero-anastomosis (insert); the latter may be preferred in the case of a congested or edematous bowel. *SB-NET* small bowel neuroendocrine tumor. Figures made in part by elements from Biorender.com

*Step 8:* The final step is the bowel resection and reconstruction. The reconstruction may be performed as a standard hand-sutured, end-to-end, one-layered anastomosis in the case of a simple wedge resection and little mesenteric involvement (Fig. 5). In cases with more extensive resection, a thick fibrous mesentery or with edematous small bowels from extensive dissection, a side-to-side anastomosis may be preferred (Fig. 5). The choice of hand-sewn over stapled anastomosis is up to the discretion of the surgeon, with extrapolation from meta-analyses showing only discrete differences between the techniques.^68,69^

**Fig. 6:**
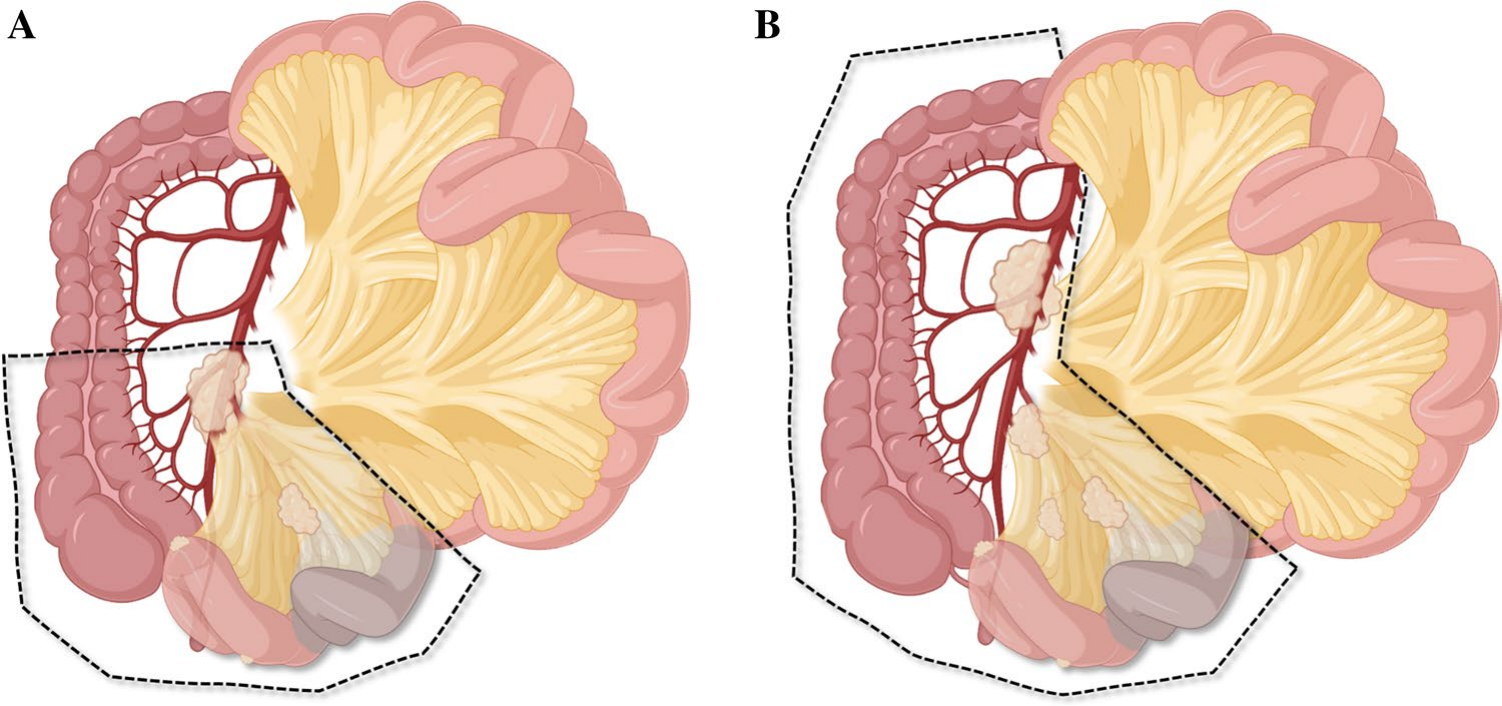
Outline of resection of an ileocecal or right hemicolectomy. Resection of the right colon [as suggested in (**A**) and (**B**)] is usually only necessary if the ileocecal artery was compromised or lesions were very close to the ileocecal valve (i.e., < 10 cm of the terminal ileum), as preservation of the ileocolic valve and colon is preferred. Figures made in part by elements from Biorender.com

Given that SB-NETs are commonly located in the terminal ileum, with over 70% located within 100 cm of the ileocecal valve,^48^ the resected bowel follows the anatomy of a right hemicolectomy (Fig. 6), although with tumors located more proximally, only the small bowel is resected and the ileocecal valve can (and should be aimed to) be preserved in the majority of cases. Only when the ileocecal artery is compromised or directly involved may a right hemicolectomy be necessary. Once the bowel is transected, it is paramount to revise hemostasis and carefully look for and, if present, fix lymphatic leaks at the root of the mesenteric transection and to align the bowel anatomically to avoid twisting or internal hernias, prior to reconstruction. Drains are not recommended or used as routine, even in extensive resections.

## Resection of Primary SB-NETs with Unresectable Liver Metastases

SB-NET primary tumors should be considered for resection, even in the presence of unresectable metastases, since primary tumors and mesenteric nodal masses left in situ may lead to mesenteric fibrosis with associated risk for complications. Indeed, resection of the primary SB-NETs despite metastatic disease has been associated with improved oncologic outcomes, mainly from reduced disease burden from mesenteric fibrosis. Good long-term survival and prevention of complications have been reported, although largely based on retrospective cohort studies.^70–72^ A meta-analysis^71^ reported a pooled hazard ratio of 0.47 (95% confidence interval 0.35–0.55) for upfront primary tumor resection compared with no resection for stage IV SB-NETs based on data from six studies. Furthermore, a population-based observation found that upfront resection of SB-NETs was associated with a reduction in unplanned acute care admissions and receipt of subsequent small bowel-related surgeries, compared with an initial strategy of non-operative management.^70^ Hence, there may be benefits of primary tumor resection in SB-NETs with unresectable metastases in order to avoid locoregional complications for a cancer that presents with a chronic course of disease. This is further emphasized in the 2017 ENETS and NANETS consensus statements, which both recommend resection of the primary tumor and regional disease in this setting.^16,17^

## Open or Laparoscopic Exploration for SB-NETs

While still controversial in this setting, minimally invasive surgery, usually in the form of laparoscopic exploration and resection, has also been entertained in some series of SB-NETs over recent years. In properly staged and selected patients, this approach may have some value,^73^ but the data are based on case reports and very small series.^74–78^ An exception is a large, nationwide cohort from The Netherlands covering a period of 1 decade. In the Dutch series, 482 patients were included from 2005 to 2015, of whom 342 (71%) underwent an open SB-NET resection and 140 (29%) underwent a laparoscopic SB-NET resection.^78^ The open resection group had significantly more multifocal tumors resected (24% vs. 14%), more pN2 lymph nodes (18% vs. 7%), and more often stage IV disease (36% vs. 22%) compared with the laparoscopic group.^78^ Hence, the more favorable patients were selected for the minimally invasive approach. The study suggested that such a strategy may be feasible in selected patients. For example, isolated lesions in or at the terminal ileum requiring an ileocecal resection or right hemicolectomy together with a distal ileum may be suitable for a laparoscopic apporach.^75,77^ However, one should recognize the risk of missing out on lesions by not performing an open exploration, as there is a high risk of additional lesions in SB-NETs (additional small intestinal NET lesions or peritoneal deposits reported in 20–30%) that are not picked up on routine imaging or even Gallium-PET scans.^79,80^ Hence, strongly recommended as the current reference approach is palpation of the small bowel that remains crucial during surgery for SB-NET. If minimally invasive surgery is employed, a bi-manual palpation of the small bowel for assessment of multifocal disease can then be facilitated through an extraction incision to run the small bowel.^73,74,76–78^

## Summary

Resection of SB-NETs should be considered in all patients, including patients with metastatic disease who are surgically fit. Key considerations include assessment of multifocality and resection of mesenteric nodal masses with the use of mesenteric-sparing approaches, and accepting of R1 margins if necessary to clear disease while avoiding short bowel syndrome. Consultation with NET-experienced surgeons should be sought prior to concluding to unresectability of locoregional disease. Decision making and management should also be tailored to patient factors, such as age and comorbidity, and goals of care.
